# Supplementary material for: Enhanced electrocaloric efficiency via energy recovery
Source: Nat Commun. 2018 May 8;9:1827. doi: 10.1038/s41467-018-04027-9 (PMC5940869; doi:10.1038/s41467-018-04027-9)
Supplement: Supplementary file 1 — Supplementary Information [file 41467_2018_4027_MOESM1_ESM.pdf]

Supplementary Information

Enhanced electrocaloric efficiency via energy recovery

E. Defay *et al.*

Supplementary Note 1

External and internal thermal time constants τ_{th}^{int} and τ_{th}^{ext} for MLCs

The initial data from Fig. 4b in the main paper are replotted below with the time axis expanded. EC heating (Supplementary Fig. 1a) is consistent with the finite element analysis (FEA) prediction of $\tau_{th}^{int} \sim 0.2$ s [S. Crossley, J. R. McGinnigle, S. Kar-Narayan and N. D. Mathur, *Appl. Phys. Lett.* **104** (2014) 082909].

Heat subsequently leaks over $\tau_{th} \sim \tau_{th}^{ext} \sim 8$ s >> τ_{th}^{int} (Supplementary Fig. 1b), where $\tau_{th} = \tau_{th}^{int} + \tau_{th}^{ext}$. Hence our ~30 s wait between transfers corresponds to ~$3.75\tau_{th}^{ext} \sim 3.75\tau_{th}$.

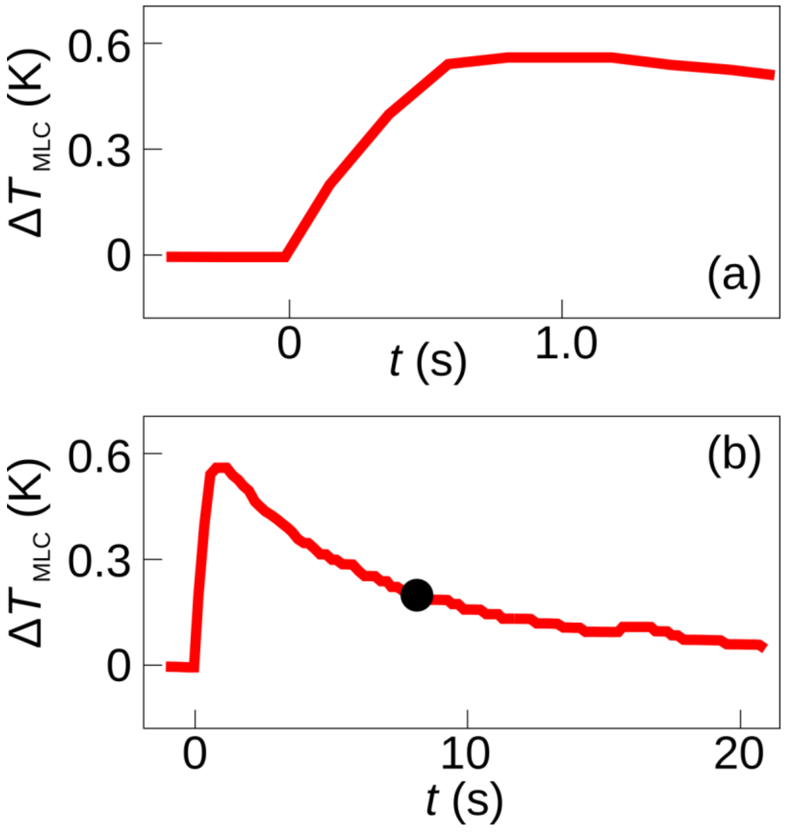

Supplementary Figure 1. EC temperature change in C1. Selected data from Fig. 4b in the main paper are plotted here to show in more detail (a) EC heating and (b) the subsequent leak of heat (black dot denotes 1/e decay with respect to the maximum temperature).

Supplementary Note 2

Maximum current during charge transfer between C1 and C2

Below we show the maximum current I_{max} during transfer i, as calculated from $E = ½LI_{max}^2$, where E is the energy to be transferred and L is the inductance of the inductor.

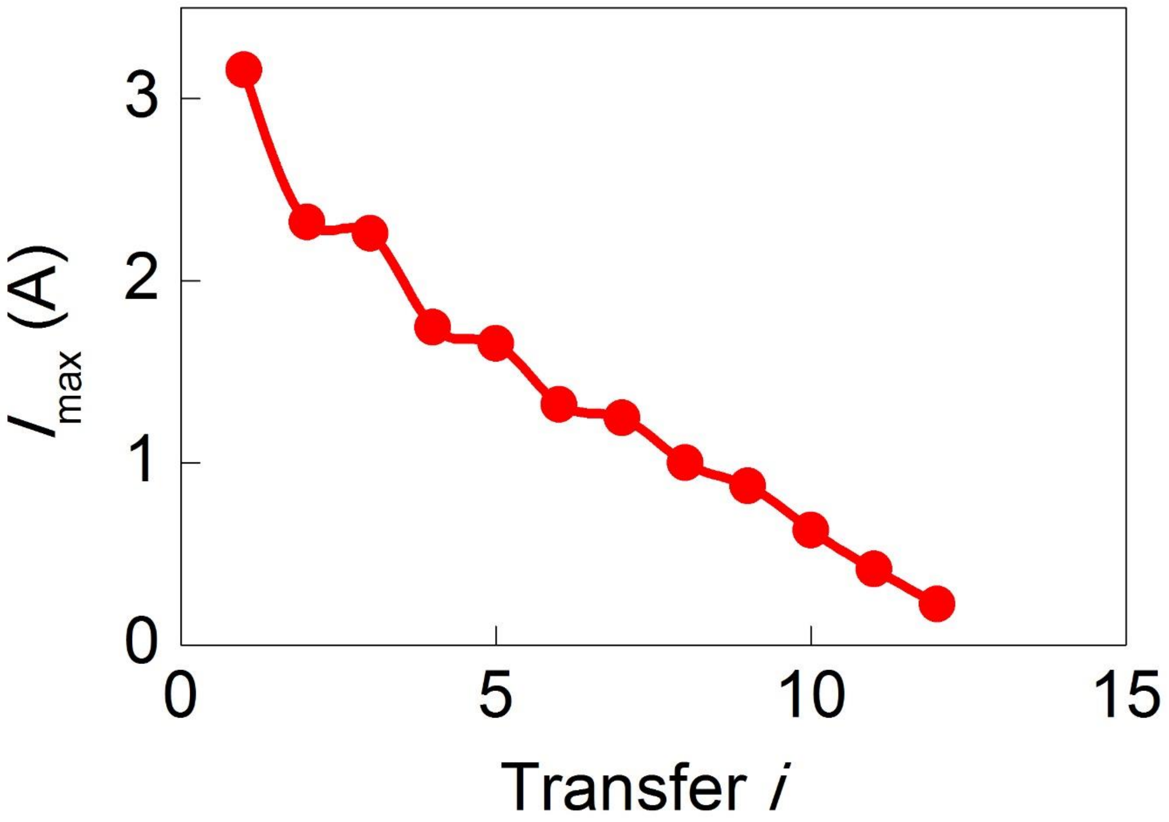

Supplementary Figure 2. Maximum current I_{max} during transfer i.

Supplementary Note 3

η^{Σ}/η_1 ~ 5 implies *WR* ~ 80%

We have:

$\eta^{\Sigma}/\eta_1 = \Sigma_{i=1}^{i=11}|Q_i/Q_1|$ from the main paper.

$= \Sigma_{i=1}^{i=11}|\eta_i W_i / \eta_1 W_1|$ as $\eta_i = |Q_i/W_i|$.

$= \Sigma_{i=1}^{i=11}|W_i / W_1|$ assuming η_i to be invariant from transfer to transfer.

Assuming that the same fraction of $W_{\text{rec}} = W_{i+1}/W_i$ of work done in each transfer is recovered for doing work in the next transfer, we may write:

$|W_1|$
$|W_2| = WR|W_1|$
$|W_3| = WR^2|W_1|$
$|W_i| = WR^{i-1}|W_1|$

Therefore:

$\eta^{\Sigma}/\eta_1 = \Sigma_{i=1}^{i=11}|W_i / W_1|$ from above becomes:

$\eta^{\Sigma}/\eta_1 = \Sigma_{i=1}^{i=12} WR^{i-1}$

$\therefore$ $5 = (1 - WR^{12})/(1 - WR)$

$\Rightarrow$ $5 \sim 1/(1 - WR)$

$\Rightarrow$ $WR \sim 0.8$

Hence *WR* ~ 80% of the work done in one transfer is recovered for the next transfer.

Supplementary Note 4

Approximate equivalence of η_i^{Δ}/η_i and η^{Σ}/η_1

The expression for $\eta_i^{\Delta}/\eta_i = W_i/(W_i - W_{i+1})$ is assumed to be independent of the value of i, so the equivalence between η_i^{Δ}/η_i and η^{Σ}/η_1 may be established for $i = 1$ by showing:

$$\eta_1^{\Delta}/\eta_1 = \eta^{\Sigma}/\eta_1$$

and hence: $$\eta^{\Sigma} = \eta_1^{\Delta}$$

Substituting with $\eta^{\Sigma} = \Sigma_{i=1}^{i=11}|Q_i/W_1|$ and $\eta_i^{\Delta} = |Q_i/(W_i - W_{i+1})|$ from the main paper, we have:

$$\Sigma_{i=1}^{i=11}|Q_i/W_1| = |Q_1/(W_1 - W_2)|$$

To show that this is true, let us make two assumptions.

First, we will assume that the same fraction of $WR = W_{i+1}/W_i$ of the work done in each transfer is recovered for doing work in the next transfer, such that:

$$|W_1|$$
$$|W_2| = WR|W_1|$$
$$|W_3| = WR^2|W_1|$$
$$|W_i| = WR^{i-1}|W_1|$$

Second, we will assume that $|Q_i| \propto |W_i|$ [by combining the good approximation $|Q^{BTO}| \propto |\Delta T^{MLC}|$ (see Methods in the main paper) with the crude approximations $|\Delta T^{MLC}| \propto |\Delta V|$ (inset, Fig. 4b in the main paper) and $|\Delta V| \propto |W|$ (Fig. 2 in the main paper)], such that $|W_i| = WR^{i-1}|W_1|$ from above becomes:

$$|Q_i| = WR^{i-1}|Q_1|.$$

The equation we are trying to show ($\Sigma_{i=1}^{i=11}|Q_i/W_1| = |Q_1/(W_1 - W_2)|$) then becomes:

$$|Q_1|\,\Sigma_{i=1}^{i=12}\, WR^{i-1}/|W_1| = |Q_1/(W_1 - W_2)|$$

Cancelling the factors of $|Q_1|$, and multiplying through by $|W_1|$ yields:

$$\Sigma_{i=1}^{i=12}\, WR^{i-1} = |W_1/(W_1 - W_2)|$$

$$\therefore \quad (1 - WR^{12})/(1 - WR) \sim 1/(1 - |W_2/W_1|)$$

$$\therefore \quad 1/(1 - WR) \sim 1/(1 - WR)$$

QED

Supplementary Note 5

Photograph of prototype cooling device based on 12C1 and 12C2

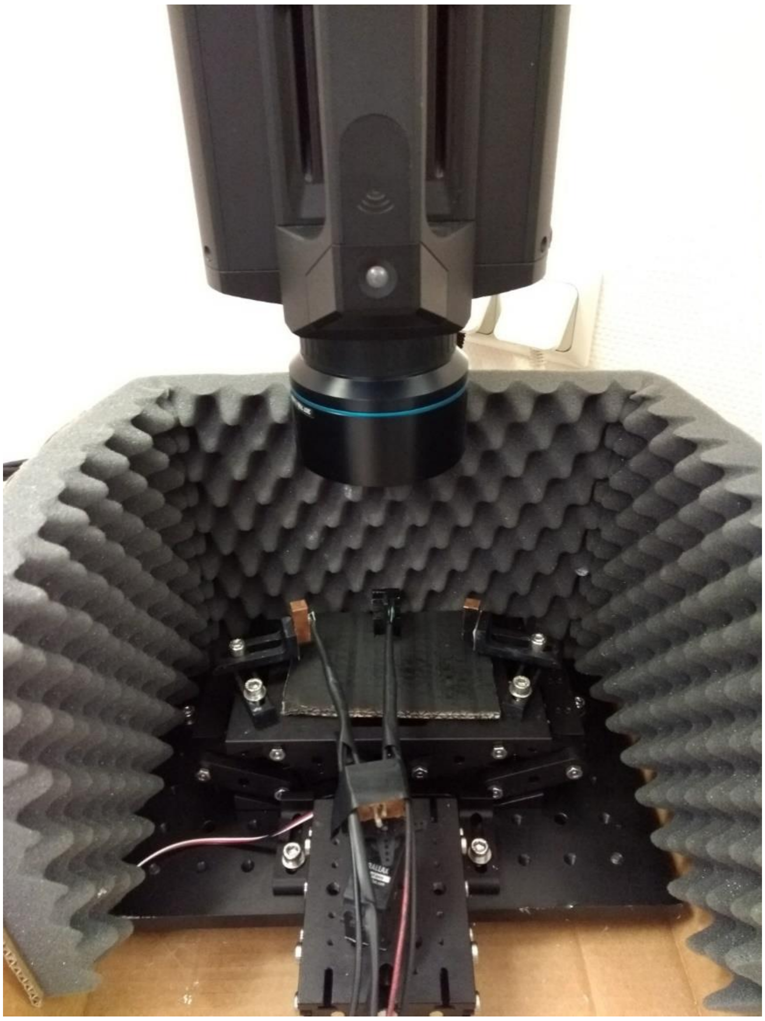

Supplementary Figure 3. Photograph of prototype cooling device. The two arms bearing MLC plates 12C1 and 12C2 appear black. The two large copper sinks sit either side of the copper heat load, whose top surface was painted black for measurements using the IR camera (partially visible at top).

Supplementary Note 6

Circuit diagram for the prototype cooling device

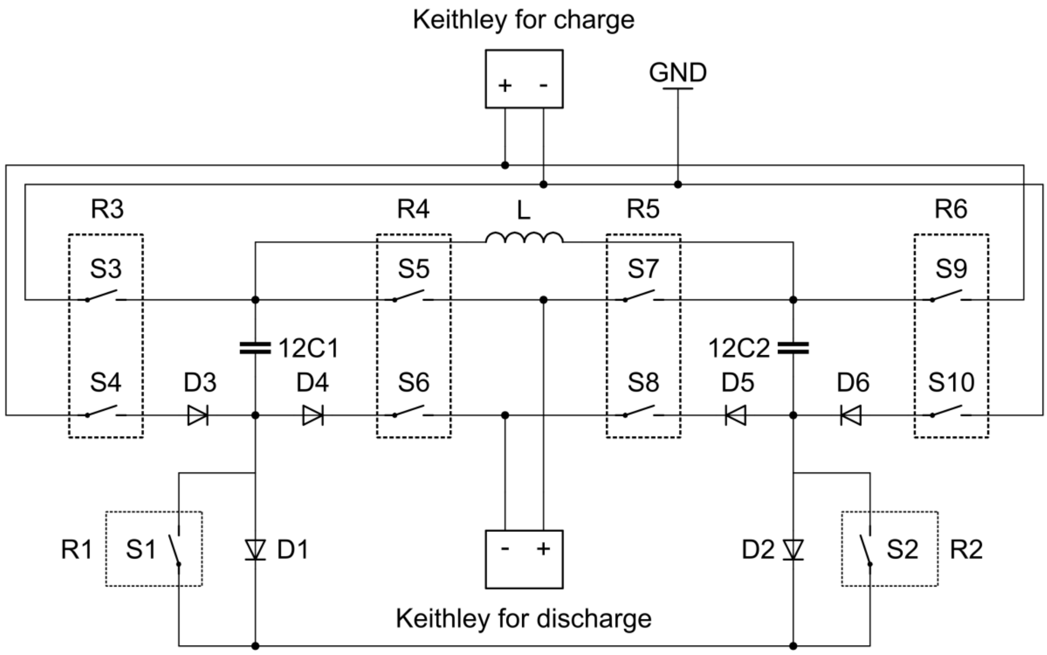

Supplementary Figure 4. Circuit diagram for the prototype cooling device. Charge transfer between EC working bodies 12C1 and 12C2 occurred via inductor L and diode D1 or diode D2 (cf. Fig. 3 of the main paper). The working body that thus received charge was then fully charged to 70 V using the upper Keithley sourcemeter via diode D3 or D6. The other working body that thus donated charge was then fully discharged using the lower Keithley sourcemeter via diode D4 or D5. Switches S1-S10 were operated by relays R1-6 (dotted lines).

Supplementary Note 7

Work done to charge 12C1 and 12C2 at the operating current

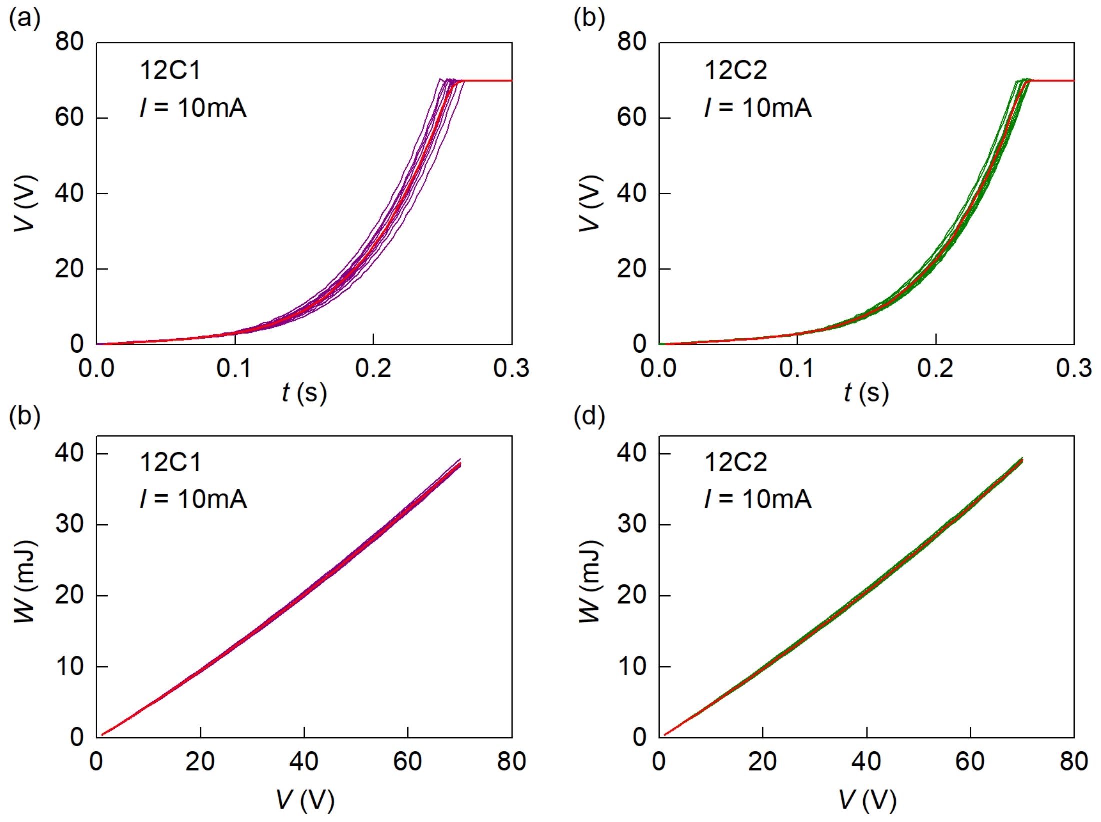

Supplementary Figure 5. Work done to charge 12C1 and 12C2 at 10 mA. For (a,b) 12C1 (purple data) and (c,d) 12C2 (green data), we present (a,c) ten measurements of voltage V versus time t and hence (b,d) the corresponding plots showing the electrical work $W(V_1 \rightarrow V_2) = I \int_{t(V_1)}^{t(V_2)} V(t')\mathrm{d}t'$ done to change the plate voltage from V_1 to V_2. Averages are shown in red were used to evaluate values of work in the main paper. Maximum voltage = 70 V.

Supplementary Note 8

Voltages across 12C1 and 12C2 during prototype operation with energy recovery

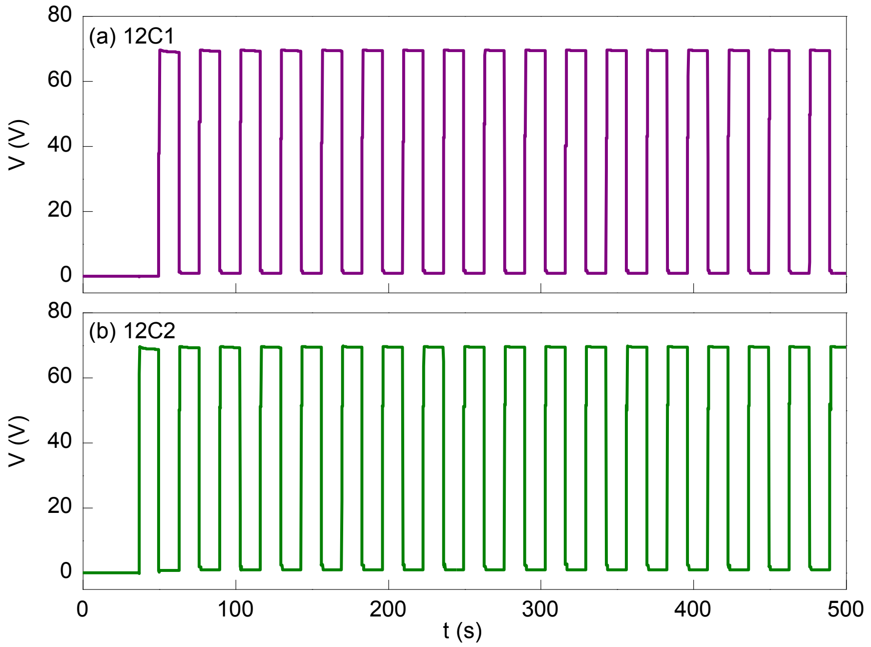

Supplementary Figure 6. Plate voltage profile during prototype operation with energy recovery. Voltage V versus time t for (a) 12C1 and (b) 12C2, showing antiphase two-step charging ($0 \rightarrow V_{hat} \rightarrow 70$ V) and two-step discharging ($70\ \mathrm{V} \rightarrow V_{lat} \rightarrow 0$) (detail for 12C2 in Supplementary Fig. 7). Data obtained while measuring load and plate temperatures (Fig. 5d in the main paper). Values of V_{hat} from here were used to identify the work $W(V_{hat} \rightarrow 70\ \mathrm{V}) = I \int_{t(V_{hat})}^{t(70\ \mathrm{V})} V(t')\mathrm{d}t'$ done in each half cycle (Fig. 5e in the main paper) via Supplementary Note 7. The intended voltage limits of 0 and 70 V were in practice 0.9 V and 69.6 V to avoid overshoot.

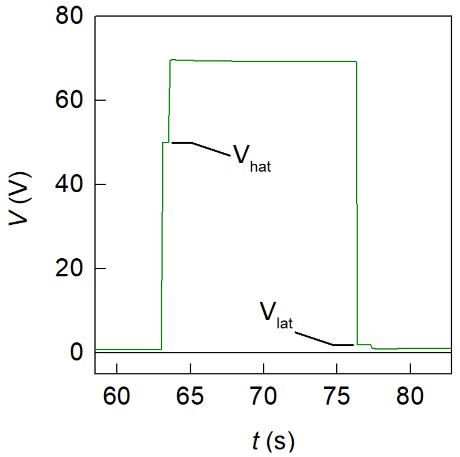

Supplementary Figure 7. Detail of plate voltage profile in Supplementary Fig. 6(b). Voltage V versus time t for 12C2 as it underwent two-step charging ($0 \rightarrow V_{hat} \rightarrow 70$ V) followed by two-step discharging ($70\ \mathrm{V} \rightarrow V_{lat} \rightarrow 0$), with a relatively long intervening period for heat exchange. The intended voltage limits of 0 and 70 V were in practice 0.9 V and 69.6 V to avoid overshoot.

Supplementary Note 9

Magnitude of the two cooling steps while modifying prototype performance

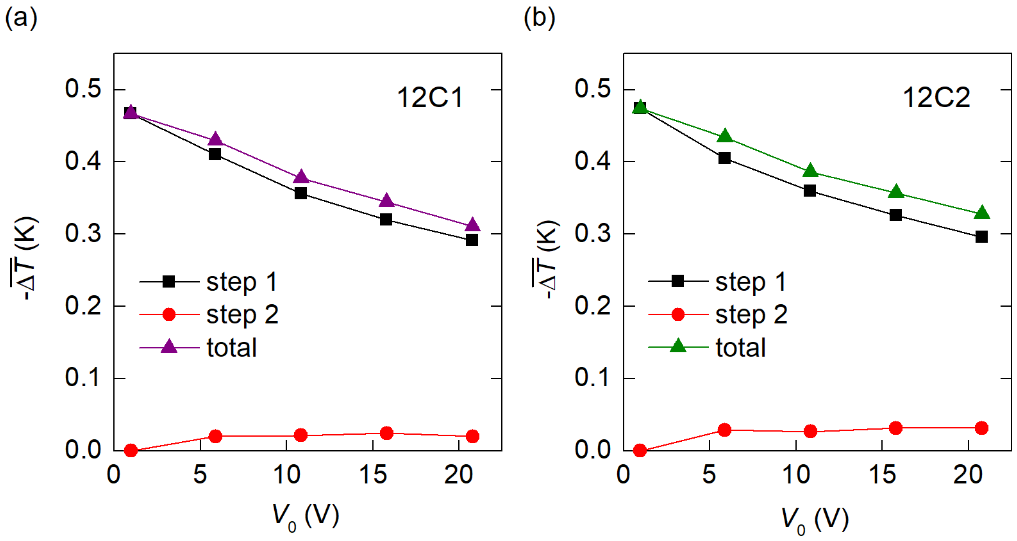

Supplementary Figure 8. Magnitude of the two cooling steps while modifying prototype performance. For (a) 12C1 and (b) 12C2, we present average values for the negative temperature change in each of the two steps -$\overline{\Delta T}$ as a function of V_0, and the resulting sum total. Data were obtained from Fig. 6a of the main paper, and averaged during the steady-state operation shown via Fig. 6b of the main paper. The intended value of $V_0 = 0$ was in practice 0.9 V to avoid overshoot.

Supplementary Note 10

Voltages across 12C1 and 12C2 during prototype operation while modifying energy recovery

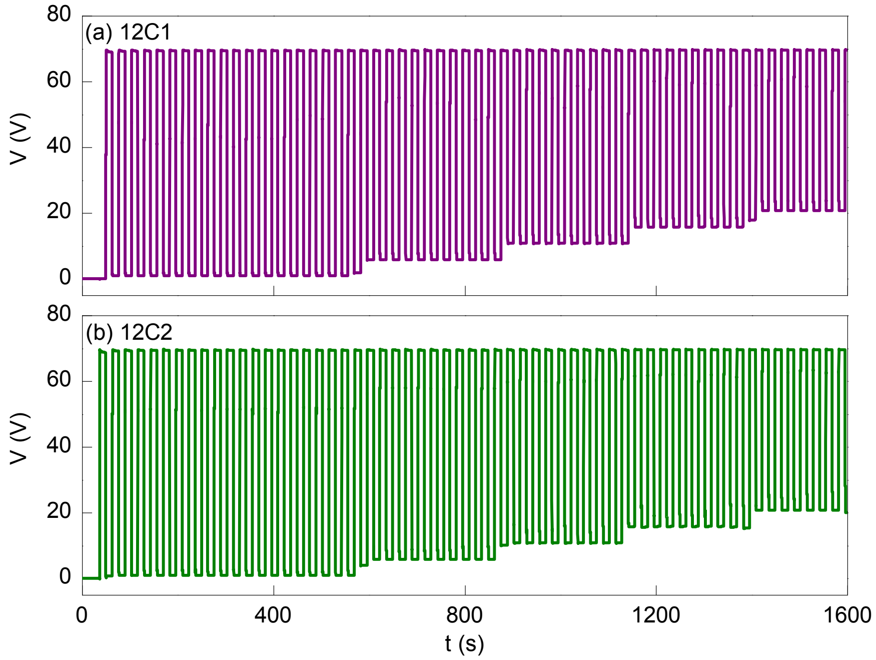

Supplementary Figure 9. Plate voltage profile during prototype operation while modifying energy recovery. Voltage V versus time t for (a) 12C1 and (b) 12C2, showing antiphase two-step charging ($V_0 \rightarrow V_{hat} \rightarrow 70$ V) and two-step discharging (70 V $\rightarrow V_{lat} \rightarrow V_0$) for different values of V_0 (detail for 12C2 in Supplementary Fig. 10). Data obtained while measuring load and plate temperatures (Fig. 6a in the main paper). Values of V_{hat} from here were used to identify the work $W(V_{hat} \rightarrow 70\text{ V}) = I\int_{t(V_{hat})}^{t(70\text{ V})} V(t')\mathrm{d}t'$ done in each half cycle (Fig. 6c in the main paper) via Supplementary Note 7. The intended voltage limits of 0 and 70 V were in practice 0.9 V and 69.6 V to avoid overshoot. On starting, $V_0 = 0$ prior to initial steady-state operation.

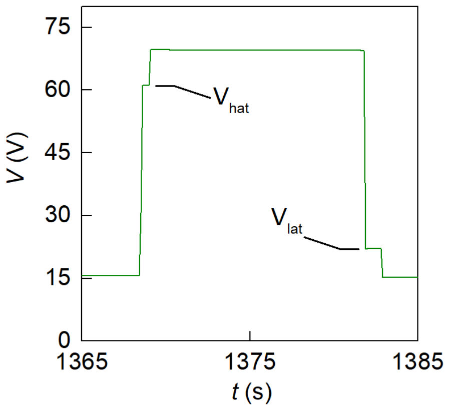

Supplementary Figure 10. Detail of plate voltage profile in Supplementary Fig. 9(b). Voltage V versus time t for 12C2 as it underwent two-step charging ($V_0 \rightarrow V_{hat} \rightarrow 70$ V) followed by two-step discharging (70 V $\rightarrow V_{lat} \rightarrow V_0$), with a relatively long intervening period for heat exchange. The intended voltage limit of 70 V was in practice 69.6 V to avoid overshoot.

Supplementary Note 11

Distinction between 12C1 and 12C2 while modifying prototype performance

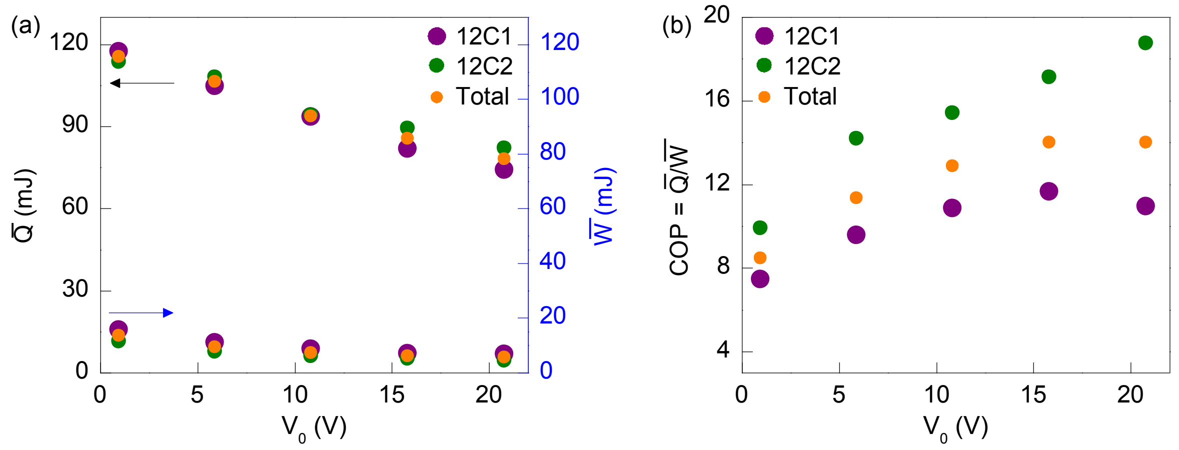

Supplementary Figure 11. Distinction between the two plates while modifying prototype performance. (a) The average heat $\overline{Q}$, the average work $\overline{W}$, and (b) the resulting COP = $\overline{Q}/\overline{W}$ as a function of V_0, presented for each plate prior to averaging in order to yield the corresponding data for the prototype (Fig. 6c,d of the main paper). Data for calculating $\overline{Q}$ were obtained from Fig. 6a of the main paper by averaging during the steady-state operation shown via Fig. 6b of the main paper. Data for calculating $\overline{W}$ were obtained from values of V_{hat} (Supplementary Figs 9 & 10) via $W(V_{\mathrm{hat}} \rightarrow 70\ \mathrm{V}) = I \int_{t(V_{\mathrm{hat}})}^{t(70\ \mathrm{V})} V(t')\mathrm{d}t'$ (Supplementary Note 7).
