# Supplementary material for: Enhanced electrocaloric efficiency via energy recovery
Source: Nat Commun. 2018 May 8;9:1827. doi: 10.1038/s41467-018-04027-9 (PMC5940869; doi:10.1038/s41467-018-04027-9)
Supplement: Supplementary file 3 — Description of Additional Supplementary Files [file 41467_2018_4027_MOESM3_ESM.docx]

**Description of Additional Supplementary Files**

File Name: Supplementary Movie 1

Description: The IR camera data show device start-up. Plates 12C1 and 12C2 take it in turns to make contact with the load, undergo adiabatic electrocaloric cooling (70 V → 0 V), and absorb heat from both the load and the voltage leads. Away from the field of view, each plate makes contact with its sink and dumps heat. The load is eventually seen to reach a steady-state temperature that is limited by heat leaks. Colour overlays denote the regions whose average temperature we report. The movie was obtained without energy recovery, but may be taken to represent device start-up with energy recovery while *V*_0_ = 0, given that the heat pumped per half cycle and the load temperature are equivalent (Fig. 5b,d).
